# How Dutch orthopedic healthcare professionals perceive antibiotic resistance: A mixed-methods application of the mental model approach

**DOI:** 10.1177/13591053251332101

**Published:** 2025-04-28

**Authors:** Lieve Vonken, Gert-Jan de Bruijn, Stef Kremers, Francine Schneider

**Affiliations:** 1 Maastricht University, The Netherlands; 2 University of Antwerp, Belgium

**Keywords:** antibacterial resistance, antibiotic resistance, attitude, beliefs, clinician, hospital personnel, perception, risk

## Abstract

Healthcare professionals must act to curb antibiotic resistance (ABR), one of today’s greatest threats to global health. This study applied the mental model approach to understanding perceptions of ABR among different Dutch orthopedic healthcare professionals. The expert model (step 1) was based on evidence-based ABR information and expert interviews (*n* = 3). This model prompted the step 2 questionnaire to inquire about perceived causes, consequences, and actions related to ABR (open-ended, *n* = 12). In the step 3 questionnaire (Likert-scales, *n* = 55), participants rated the impact of causes of ABR, the likelihood and severity of consequences of ABR, and the effectiveness of actions against ABR. Step 3 showed that no specific causes, consequences, or actions are perceived to strongly outweigh the others. Dutch orthopedic healthcare professionals perceive the causes of ABR to be mostly external, the consequences of ABR to be abstract and the most effective actions against ABR to be performed by others.

## Introduction

When bacteria are no longer affected by antibiotics this is called antibiotic resistance (ABR; World Health Organization, 2023). ABR directly results in infection-related morbidity and mortality since resistant bacteria are much more difficult and sometimes impossible to treat and ABR may increase the risk of adverse effects in procedures that rely on antibiotics, resulting in these procedures being performed less often (Naylor et al., 2018). This applies to, for example, surgeries using prophylactic antibiotics, like many orthopedic surgeries (Stichting Werkgroep Antibioticabeleid, 2019). Estimations have associated ABR with 4.95 million deaths in 2019 (Murray et al., 2022) and the WHO considers ABR one of the greatest threats to global health, food security, and development today (World Health Organization, 2023).

Almost all causes of the ABR crisis can be linked back to behavior: healthcare professionals prescribe antibiotics inappropriately, patients overuse antibiotics, as do agriculturists, and pharmaceutics develop insufficient new antibiotics (Ventola, 2015). Following the high amounts of antibiotics used in healthcare, and the role of healthcare professionals as gatekeepers to these antibiotics, healthcare professionals’ responsible behavior regarding ABR is paramount (de Greeff et al., 2023).

Antibiotic stewardship programs are among the most prominent initiatives against ABR directed at healthcare professionals (Pollack and Srinivasan, 2014). However, these programs are only mildly effective in promoting responsible behavior and curbing ABR (Bertollo et al., 2018). To improve this, it is argued that more research should be directed toward inducing behavior change at the individual level (van Den Bergh and Brink, 2021). Specifically, many potential lies in social and behavioral theories and tools which have proved their value in numerous other fields, where they have been used to explain and change various behaviors (Hawkins et al., 2023; Hemenway and DuBois, 2022). For example, behavioral insights have effectively been applied in healthcare quality improvement (Johansen and Tulloch, 2024). Still, social and behavioral science insights are applied scarcely and often inadequately in promoting healthcare professionals to act against ABR (Hawkins et al., 2023; Hemenway and DuBois, 2022). Behavior change theories assume that behavior is caused by determinants and the beliefs influencing these determinants. To induce behavior change, specific determinants and their underlying perceptions should be targeted (Metz et al., 2022). Consequently, to apply behavior change tools effectively detailed perceptions must be known.

Previous studies have identified perceptions related to ABR that might explain healthcare professionals’ current behavior (Vonken et al., 2024). Findings of these types of studies, for example, indicate that although healthcare professionals recognize the potential consequences of ABR, they are unaware of the current scale of the problem, perceive the consequences of ABR as abstract and in a distant future, and doubt their susceptibility (Krockow et al., 2019; Vonken et al., 2024). Moreover, healthcare professionals perceive their influence on curbing ABR as limited and their potential behavior change as futile (Keizer et al., 2019; Krockow et al., 2019). However, as is the case for all human behavior, behaviors related to ABR are generally not influenced by a few specific, simple perceptions, but rather by complex combinations of many perceptions of varying importance (Samvel et al., 2020). For example, antibiotic prescribing behavior, one of the largest contributors to ABR, is largely influenced by perceptions of direct risk for patients, but also by perceptions of ABR (Warreman et al., 2019). When prescribing antibiotics, healthcare professionals might perceive the drawback of potentially increasing ABR not to outweigh the benefit of more certainly curing the patient (Warreman et al., 2019). To fully understand healthcare professionals’ behavior related to ABR, a comprehensive understanding of healthcare professionals’ perceptions of ABR and the relative importance of these perceptions is needed. Although relevant perceptions have been identified before, synthesizing perceptions from existing studies into a comprehensive overview is challenging, because this would require rating the relative importance of perceptions identified in different studies, with different methodologies and target groups. The difficulty of combining results from multiple studies is increased because it is often unclear which constructs are measured and rationales for operationalization of questions are deficient (Vonken et al., 2024). To create a comprehensive overview of healthcare professionals’ perceptions, one study investigating a broad range of perceptions with a consistent methodology is needed instead of different studies investigating different perceptions with different methodologies.

This study aims to create such a comprehensive overview of perceptions by zooming in on orthopedic healthcare professionals. Orthopedic procedures are often highly invasive, applying foreign materials (prostheses) that remain in the body for many years. This results in a high infection risk (Li et al. 2013). When infections do occur, this severely worsens patients’ health-related quality of life and life expectancy. (Wildeman et al., 2021) To manage infection risk, orthopedic procedures strongly rely on prophylactic antibiotics. (Stichting Werkgroep Antibioticabeleid, 2019) Because of the high infection risk, the severe consequences of infection, and the large dependence on (prophylactic) antibiotics, the consequences of ABR for orthopedics are potentially catastrophic. This makes the field of orthopedics an interesting case study.

This study aims to create a comprehensive overview of orthopedic healthcare professionals’ perceptions of ABR. Such a combination of perceptions can be called a mental model. Mental models include internal representations, simplified ideas, of “how things work” that people use to make sense of the world around them and are rarely solely based on facts (Johnson-Laird, 2010; Morgan, 2002). The mental model approach is a method to map perceptions in a model and ultimately, develop risk communication that fits this model (Morgan, 2002). This approach has been used to investigate perceptions about COVID-19 (de Ridder et al., 2022; Greenhalgh, 2021) and healthcare professionals’ perceptions of bacterial biofilms (Bjarnsholt et al., 2021). The exploratory first steps of the mental model approach make it especially useful for understanding perceptions when previous research is lacking or paints an unclear picture. The following steps will support the identification of perceptions in a complete and detailed way. Knowing these perceptions will inform future studies aiming to promote healthcare professionals’ rational behavior regarding ABR (Li and Webster, 2018).

## Methods and results

Our design was based on the first three steps of the mental model approach as described by Morgan: creating an expert model, conducting mental model interviews, and conducting structured interviews (Morgan, 2002). Considering healthcare professionals’ sparse time, we conducted questionnaires instead of interviews in steps 2 (open-ended) and 3 (multiple choice). Ethical approval for this study was received from the Maastricht University Faculty of Health, Medicine and Life Sciences Research Ethics Committee (FHML-REC/2022/097).

### Step 1: Creating an expert model

#### Design

The first draft of the expert model (influence diagram) was based on evidence-based ABR information; a Dutch antimicrobial resistance webpage with evidence-based information about public health and care, developed by the National Institute for Public Health and the Environment (https://www.vzinfo.nl/antimicrobi%C3%ABle-resistentie-amr/gevolgen) and the European Centre for Disease Prevention and Control antimicrobial resistance webpage (https://www.ecdc.europa.eu/en/antimicrobial-resistance). This draft was then improved in expert discussions. In these discussions we applied a combination of the exposure-to-effects method (i.e. working from a template based on the biological mechanism) and the assembly method (i.e. listing all relevant factors and then figuring out how they are related). Exposure-to-effects, in this case, meant that the model contained causes, consequences, and actions related to ABR in this order. The assembly method was applied within these three categories. In all expert discussions, the experts were told that this model should show orthopedic healthcare professionals’ perceptions about ABR and that it will ultimately be used to develop ABR risk communication. Experts were shown three example expert models to ensure their understanding of our question to them (Appendix A).

#### Participants and recruitment

Experts were purposefully recruited through the network of the research team (LV, GJdB, SK, FS). Experts who had experience with orthopedics in the Netherlands were recruited to reflect expertise in combatting ABR, new methods for combatting ABR in orthopedics, and infection prevention. We included an expert in hospital infection prevention and control, an expert in (technology for) orthopedic infections, both employed at the Maastricht University Medical Centre (The Netherlands), and an expert in different methods for curbing ABR. Moreover, the model was checked with a presentation on this topic from an orthopedic surgeon who specializes in infections.

#### Results

**Figure 1.**
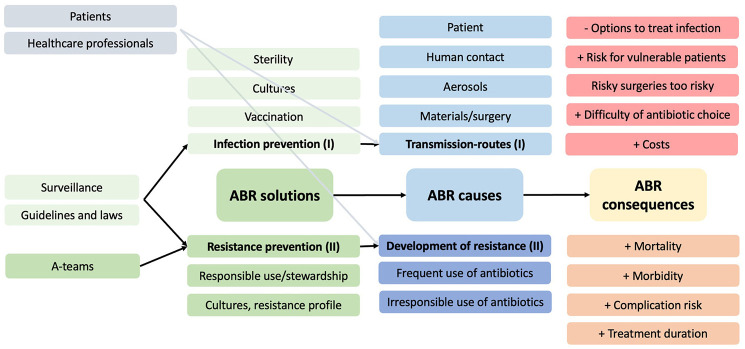
Final expert model, result of step 1. This is a simplified version of the model. The model constructed with the experts can be found in Appendix B on OSF: https://osf.io/dkty3/ .

In their answers, all experts adhered to the structure of causes, consequences, and actions that followed the exposure-to-effects method. As a result of the assembly method, the infection prevention specialist suggested causes and solutions related to infection prevention (e.g. patient factors) and the expert on action against ABR added alternative solutions (e.g. the vaccination of healthcare personnel). The other sources were used to specify existing topics in the model (e.g. adding *predisposing factors* to the patient factors causing ABR). All three experts indicated that the model they were presented with was a good representation of ABR causes, consequences, and solutions. A simplified and English version of the expert model is displayed in Figure 1.

### Step 2: Mental model open-ended questionnaire

#### Design

Step 1 yielded an expert model, or influence diagram, including causes, consequences, and actions related to ABR. Based on this model, a questionnaire was developed for step 2 (Table 1; Appendix C on the Open Science Framework [OSF]: https://osf.io/dkty3/). The first part of the step 2 questionnaire consisted of descriptive items. The second part of this questionnaire consisted of items covering perceptions related to ABR. Based on step 1, causes, consequences, and actions related to ABR were chosen as the most important topics to address. For all three categories (causes, consequences, and actions) we first asked them to list all they could think of. Then, we provided them with their answers and asked for follow-up items.

**Table 1.** Content of the step 2 questionnaire.

Question		Answer options
Descriptive		
Type of organization		Multiple choice with “other” option
Job description		Multiple choice with “other” option for type of medical professional (i.e. medical doctor), and free input for specialty (i.e. orthopedics)
Years of experience		Multiple choice [1–50]
How often they work on ABR		Multiple choice [5-point likert; daily—less than half-yearly]
Whether they consider themselves an ABR expert		Multiple choice [7-point likert; strongly disagree—strongly agree]
Perceptions related to ABR		
What they believe healthcare professionals should know about ABR	The influence on the hospital context and	Open-ended
	The influence outside this context	Open-ended
Causes	List all causes they can think of	Open-ended, multiple answers possible
	Who they think is responsible for this cause	Open-ended
Consequences	List all consequences they can think of	Open-ended, multiple answers possible
	Who they think will be affected	Open-ended
	How severe this consequence is	[7-point likert; not at all severe—very severe]
	How likely this consequence is within 6 months	[7-point likert; very unlikely—very likely]
	How likely this consequence is within 10 years	[7-point likert; very unlikely—very likely]
Actions	List all actions they can think of	Open-ended, multiple answers possible
	How effective this action is	[7-point likert; very ineffective—very effective]
	How feasible this action is	[7-point likert; very unfeasible—very feasible]
	What barriers and facilitators would be	Open-ended

To ensure that no relevant topics were missed in step 2, we compared the results of step 2 with the expert model and, in addition, with previous literature about perceptions of healthcare professionals about ABR. This extra step was performed to supplement the answers to the open-ended questionnaire because the interviews recommended by Morgan would possibly have yielded even richer data (Morgan, 2002). Six diverse studies on this topic were chosen for comparison from a recent systematic review (Alothman et al., 2016; Alsayed et al., 2022; Alzoubi et al., 2009; Navarro-San Francisco et al., 2013; Toska and Geitona, 2015; Wester et al., 2002). We added new studies until these no longer supplied new themes. Only in the causes category, three items were added, related to patient demands and expectations for antibiotics, self-medication, and pharmaceutical companies advertising and promoting antibiotics. The questionnaire was pilot-tested with two doctors and two researchers who did not work in orthopedics of whom one had no prior knowledge about ABR. Minor changes to the phrasing of the items were made.

#### Participants and recruitment

Participants were healthcare professionals who worked in orthopedics in the Netherlands and were recruited by inviting participants from a previous study (Vonken et al., 2024), and through the networks of project members (https://nwa-dartbac.eu). All participants read an information text and indicated informed consent. Participants were not reimbursed.

#### Data analysis

All step 2 data was prepared and analyzed in Microsoft Excel. Only responses with answers to at least one item about perceptions were analyzed. Quantitative items were summarized using percentages (nominal data), or mean, standard deviation, and range (continuous data). Content analysis was applied to the qualitative data (Hsieh and Shannon, 2005). All responses were listed and fragments were coded in an iterative process: codes and themes were sought, reviewed, and adjusted while coding.

#### Results

Responses were collected in November and December 2022. Twelve respondents filled in at least one answer category related to the research question (causes, consequences, solutions). Respondents who did not fill in any of these answer categories were excluded (*n* = 10; of whom *n* = 7 dropped out before entering any personal information or perceptions). Most respondents fully completed the questionnaire (*n* = 11). Most participants worked at an academic hospital (91.7%; *n* = 11) and 8.3% (*n* = 1) at a community hospital. Most participants consisted of medical specialists (66.7%, *n* = 8), followed by researchers (16.7%, *n* = 2), a doctor not in residency (8.3%, *n* = 1), and a physician assistant (8.3%, *n* = 1). Healthcare professionals mostly worked in orthopedics (75%, *n* = 9). Other fields included medical microbiology, implantable materials, and healthcare evaluation (all 8.3%, *n* = 1). Years of experience ranged from 1 to 19 with a mean of 9.92 years (SD 6.95). Most participants work on ABR issues at least weekly (86.3%; *n* = 8). Half of the participants indicated to be ABR experts (*n* = 6; Table 2).

**Table 2.** Characteristics of step 2 participants: ABR experience.

How often they work on ABR			Perceive themselves as ABR expert		
	*N*	%		*N*	%
Daily	3	25.0	Strongly disagree	1	8.3
Weekly	7	58.3	Disagree	2	16.7
Monthly	1	8.3	Somewhat disagree	1	8.3
Half-yearly	0	0	Neither agree nor disagree	2	16.7
Less than half-yearly	1	8.3	Somewhat agree	3	25.0
			Agree	3	25.0
			Strongly agree	0	0

When asked what they believed healthcare professionals in their position should know about the influence of ABR on processes in the hospital context, several themes emerged. Participants felt that healthcare professionals should know “how to treat ABR prudently” and should be “prepared to use alternatives to antibiotics.” In addition, they should know about the “mechanism” of ABR, “guidelines and research,” their “role in prevention,” and what the “consequences” are. They should have a general “awareness” of the problem of ABR. Moreover, they should be aware of the “limits of their knowledge.”

#### Perceptions regarding causes, consequences, and actions

When asked about ABR causes, most answers related to *healthcare professionals* and the *hospital*. “Hygiene” (in the OR), “antibiotic prescribing” (i.e. “inappropriate,” “based on wrong diagnoses,” “too much and/or too long,” “too short,” or “too little,” “too broad,” “too liberal” or “preventive”), and the “increase in implant surgery” were mentioned. Moreover, several of the mentioned causes were related to *patients* (i.e. “not following prescriptions,” “impaired immune responses,” “other medication,” “having been treated with antibiotics before,” “bringing in bacteria from other hospitals” or “abroad”). In addition, the “*changing resistance of bacteria”* and the use of *antibiotics in other countries and sectors* (i.e. “the food sector”) were mentioned.

Participants expected ABR to lead to “*increased infections and resistance,”*“*higher care costs,”* and “*increased mortality and morbidity.”* In *healthcare*, ABR was expected to lead to “difficult” or “untreatable infections” (i.e. “less,” “only back-up,” or “no antibiotics available”), a “reduction in treatment options,” “worse patient care” (i.e. “more intense treatment”or “complications”), an increased need for “isolation capacity”, “altered care deliberations” (i.e. choosing not to operate in case of high infection risk) and a “higher burden on healthcare” (i.e. “means” and ”nurses”) and “shutting down wards.”

Actions participants listed that they believe to be effective against ABR mostly related to the *healthcare context*. These actions included collaboration (i.e. in “multidisciplinary teams”), policy (i.e. “fines”), “guidelines” and “protocols,” “antibiotic stewardship” targeting “hygiene” as well as “prescribing,” “monitoring antibiotic use” and “resistance,” altering surgical procedures (i.e. “infection prevention,” using “biomaterials,” “not operating on risk groups”), “evidence-based practice,” and “education” of and “awareness among healthcare professionals.” Moreover, “*education” of and “awareness* among patients” were mentioned. In addition, participants listed “*research,” “appropriate funding”, and “prevention in other sectors”* (e.g. the “food industry”).

### Step 3: Structured rating questionnaire

#### Design

The results of step 2 informed step 3. The step 3 questionnaire consisted of descriptive items like those in the step 2 questionnaire and a second part inquiring about perceptions (Appendix C on OSF: https://osf.io/dkty3/). The three categories (consequences, causes, and actions) were now covered in items listing the summarized output of the previous questionnaire and applying Likert scales. For causes, the item applied a 7-point Likert scale to infer the extent to which something was perceived as a cause of ABR. For consequences, participants were randomized to see the items framed as consequences for themselves (condition 1) or their patients (condition 2). They were first asked to rate the likelihood of the consequences on a 7-point Likert scale, then they ranked the severity of all consequences which they ranked as >5 likely, again on a 7-point Likert scale. For actions, participants first rated the effectiveness of several actions on a 7-point Likert scale. Second, they were randomized to see the actions to be performed by themselves (condition 1) or their organization (condition 2). For all actions that they rated >4 effective, they were asked to select the action for which they had confidence in their or their organization’s ability to perform. In open-ended items, they were asked about barriers, benefits, and cues to perform these actions. To ensure that no causes, consequences, or actions were missed, participants could select “other” and add a text response containing a cause, consequence, or action at the beginning of each answer category. The questionnaire was pilot-tested with the same group as the first questionnaire. Minor changes to the phrasing of the items were made.

#### Participants and recruitment

Participants were recruited by inviting the invitees for step 2, through the newsletters of the Dutch Orthopedic Association (NOV; https://www.orthopeden.org) and the Dutch Association of Orthopedic Surgical Residents (VOCA; https://voca.org), on LinkedIn (including the page of VOCA) and by calling orthopedics departments of Dutch hospitals. All participants read an information text and indicated informed consent. The information text described the purpose of the study and the target group. When participants indicated working in a different country than the Netherlands, they were directed to the end of the questionnaire. No further inclusion criteria were applied to reflect the broad range of different healthcare professionals in orthopedics. Participants were not reimbursed.

#### Data analysis

Only responses with answers to at least one item about perceptions were analyzed. Fisher’s Exact tests (for categorical data) and a Mann-Whitney *U* test (for continuous data) were used to compare the characteristics of excluded and included participants. Descriptive items were summarized using percentages (nominal data), mean, standard deviation, and range (years), or median and interquartile range (IQR; continuous data) in SPSS 27. Items related to participants’ perceptions were visualized in (split-)violin plots with boxplots or bar charts using R 4.3.2 and RStudio 2023.09.01. The violin plots show the distribution of the responses, and the boxplots show the median, IQR, and outliers. The bar charts show the count. The agreement among the participants was determined based on the IQR (high [size ≤ 2], medium [size > 2 and < 4], or low size ≥ 4]), and the height of the median was described (low [≤2], medium-low [> 2 and < 4], medium [4], medium-high [>4 and < 6], or high ≥ 6]).

#### Results

Answers were collected from May to September 2023. Fifty-five respondents filled in at least one answer category related to the research question (causes, consequences, solutions) and fit the inclusion criteria. Respondents who did not fill in any of the answer categories related to the research question were excluded (*n* = 51; of whom *n* = 40 dropped out before entering any personal information or perceptions). Participants who dropped out most often worked in community hospitals (76.4%, *n* = 10), were medical specialists (54.6%, *n* = 6; orthopedics, *n* = 4; trauma surgery, *n* = 1; microbiology, *n* = 1) or nurses (45.5%, *n* = 5). Data on these excluded participants is available on OSF https://osf.io/dkty3/. A Fisher’s Exact test showed no association between exclusion status (being excluded or included in the analysis) and hospital type, job description, how often they work on ABR, being an ABR expert, or perceived ABR urgency. In addition, years of experience in excluded participants (median: 7; IQR 2–16) did not differ significantly from included participants (median: 4; IQR 2–10), *U* = 243.5, *z* = −1.020, *p* = 0.308.

Most respondents fully completed the questionnaire (70.9%, *n* = 39). Healthcare professionals mostly worked in community hospitals (76.4%, *n* = 42) and were medical specialists (30.9%, *n* = 17) or nurses (27.3%, *n* = 15), Table 3. Most participants worked in orthopedics (58.2%, *n* = 32; in combination with a different field such as traumatology: 7.3%, *n* = 4). Some participants (30.9%; *n* = 17) did not clearly state their field. Years of experience ranged from 1 to 39 with a mean of 7.49 years (SD 8.219). Most participants work on ABR issues at least weekly (58.2%; *n* = 32; Table 3). One-quarter of the participants indicated to be ABR experts (25.4%; *n* = 14). All participants evaluated ABR as an urgent problem. One additional cause (“behavior”) and two additional actions were mentioned (“worldwide approach needed” and “national guidelines”). However, these were evaluated as being repetitive or outside the scope of this study.

**Table 3.** Characteristics of step 3 participants: job context, ABR experience and felt urgency of ABR.

Hospital type			Job description/main purpose			How often they work on ABR		
	*N*	%		*N*	%		*N*	%
Community hospital	42	76.4	Medical specialists	17	30.9	Daily	7	12.7
			Nurses	15	27.3	Weekly	25	45.5
Academic hospital	12	21.8	Residents	7	12.7	Monthly	10	18.2
Integrated treatment center	1	1.8	Doctors not in residency	6	10.9	Half-yearly	3	5.5
			Researchers	1	1.8	Less than half-yearly	10	18.2
			Other^a^	9	16.4			
ABR expert		*N*	%		ABR urgency		*N*	%
Strongly disagree		6	10.9	Not at all urgent			0	0
Disagree		15	27.3	Not urgent			0	0
Somewhat disagree		4	7.3	Somewhat not urgent			0	0
Neither agree nor disagree		16	29.1	Neutral			0	0
Somewhat agree		11	20.0	Somewhat urgent			19	34.5
Agree		2	3.6	Urgent			32	58.2
Strongly agree		1	1.8	Very urgent			4	7.3

a Includes operating assistants (*n* = 4), physician assistants (*n* = 2), a physiotherapist (in the orthopedic department), a medical intern, and a team leader.

#### Perceptions regarding causes

**Figure 2.**
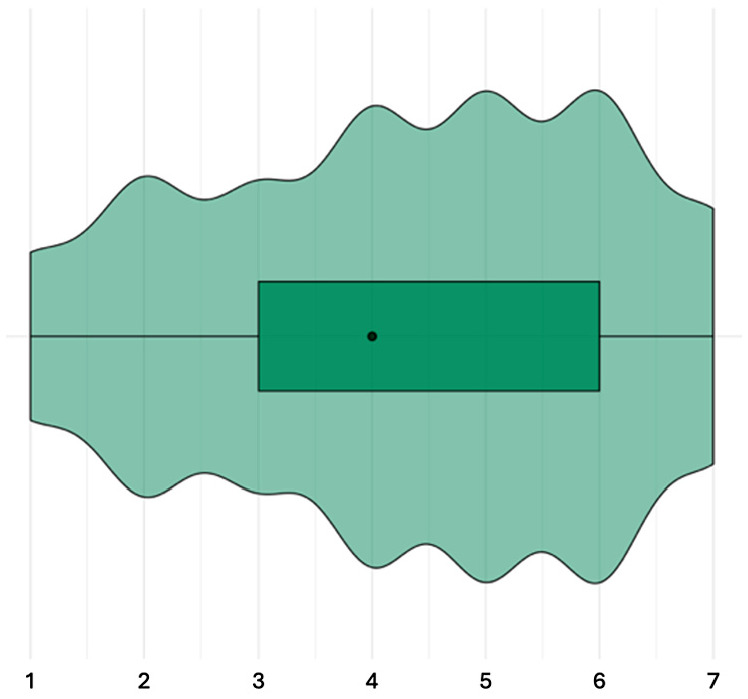
Result of step 3: perceived impact of causes of ABR, all causes aggregated. Not a cause [1]—very much a cause [7].

**Figure 3.**
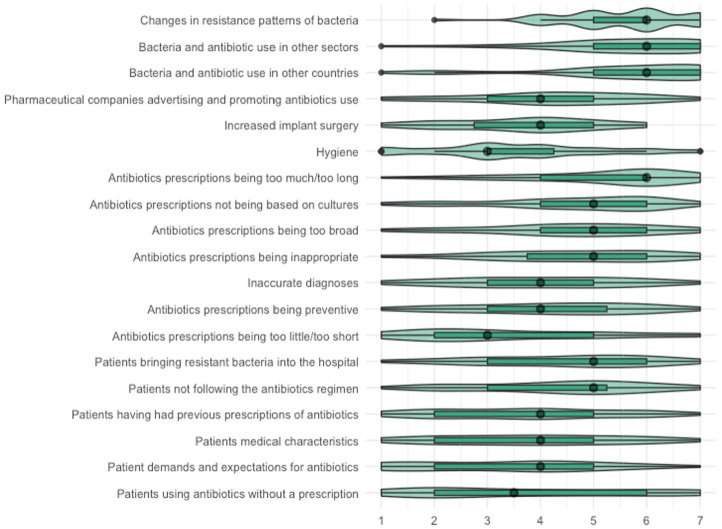
Result of step 3: perceived impact of causes of ABR, causes specified. Not a cause [1]—very much a cause [7].

The items about causes were answered by 44 participants. Overall, participants believe that the listed causes have a medium impact on ABR (Figure 2; median score of all causes combined: 4 out of 7; IQR: 3–6). Participants were in medium to high agreement for all causes unrelated to patients (IQR of size 1–3; Figure 3). For these causes, participants believed causes attributable to other countries and sectors and changing bacteria have a high impact on ABR (median: 6; IQR 5–7). They also recognized causes related to prescribing, although they rated the impact of these causes to be medium-low to medium-high. For example, prescriptions being too much or too long (median: 6; IQR 4–6) was perceived to have a higher impact than prescriptions being preventive (median: 4; IQR 3–5.25) and too little or too short (median: 3; IQR 2–5). Participants believed that most causes related to external factors, either organizational (e.g. increased implant surgery, inaccurate diagnoses) or related to pharmaceutical advertisements or preventive prescribing, have a medium impact on ABR (median: 4; IQR 2.75–5.25). The impact of hygiene on ABR was perceived to be medium-low (median: 3; IQR 3–4). For causes related to patients, the agreement among participants was medium or low (IQR of size 2.25–4). These causes were perceived to have a medium-low to medium-high impact on ABR. For example, patients using antibiotics without a prescription (median: 3.5; IQR 2–6) were perceived to have a lower impact than patients not following the antibiotics regimen (median: 5; IQR 3–5.25).

#### Perceptions regarding consequences

The items about consequences were answered by 49 participants, 23 of these were instructed to rate how likely they perceived the consequences to be for patients, and 26 were instructed to rate how likely they perceived the consequences to be for healthcare professionals. Participants rated these consequences to be medium likely for patients (median likelihood of all consequences combined: 4 out of 7; IQR: 3–5; Figure 4). Participants rated these consequences to be medium-high likely for healthcare professionals (median likelihood of all consequences combined: 5 out of 7; IQR: 4–6).

**Figure 4.**
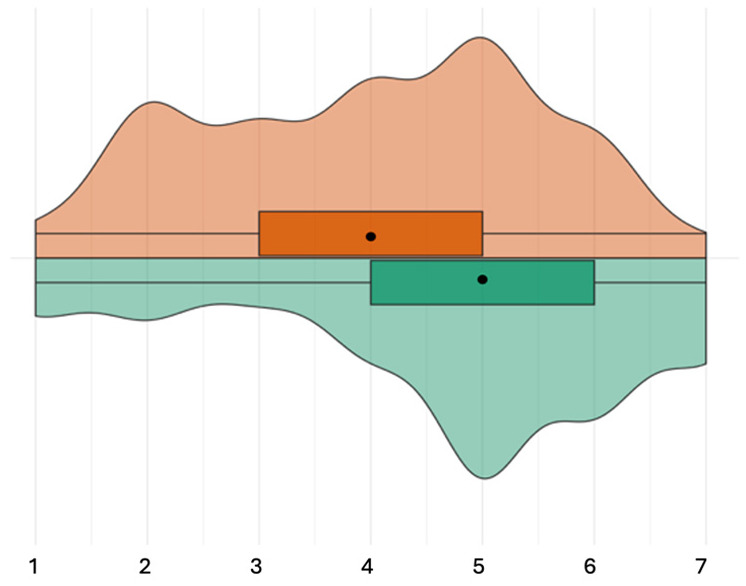
Result of step 3: perceived likelihood of consequences of ABR, all consequences aggregated. Very unlikely [1]—very likely [7], orange = patient condition, green = healthcare professional condition.

**Figure 5.**
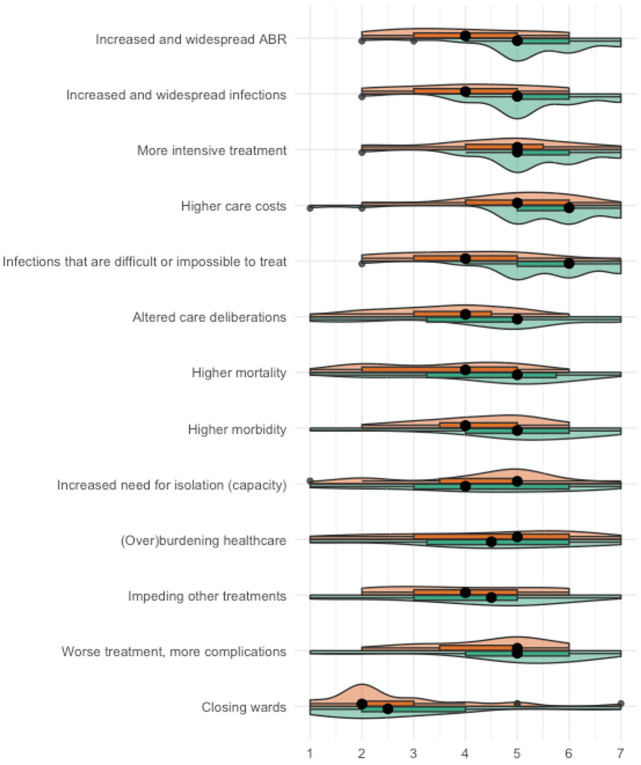
Results of step 3: perceived likelihood of consequences of ABR, consequences specified. Very unlikely [1]—very likely [7], orange = patient condition, green = healthcare professional condition.

The agreement among participants was high for 10 out of 13 consequences (IQR of size 1–2) and medium for higher mortality, increased need for isolation (capacity), and (over)burdening healthcare (IQR of size 1.5–3; Figure 5). Only in the healthcare professional condition were consequences perceived highly likely, specifically higher care costs (median: 6; IQR 5–6) and infections that are difficult or impossible to treat (median: 6; IQR 5–6). The least likely consequence was closing wards because of ABR (median patient condition: 2; IQR 2–3; median healthcare professional condition: 2.5; IQR 2–4).

#### Perceptions regarding actions

**Figure 6.**
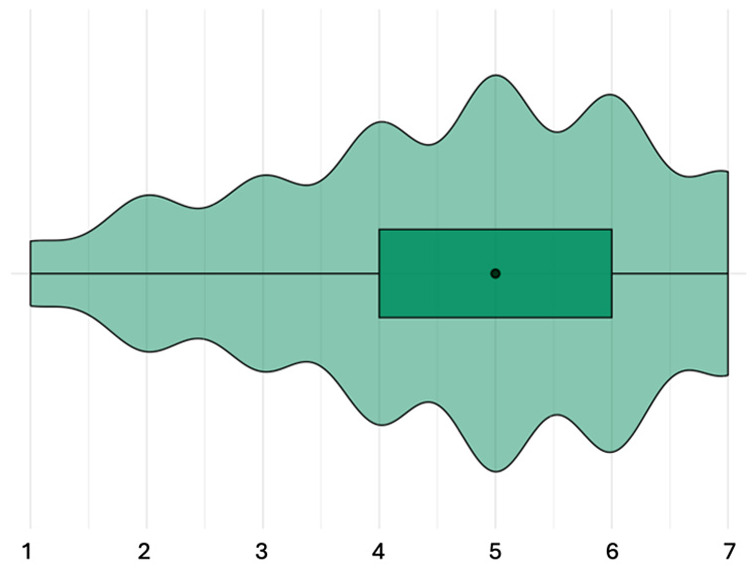
Results of step 3: perceived effectiveness of actions against ABR, all actions aggregated. Very ineffective [1]—very effective [7]).

The items about actions were answered by 45 participants. Participants believe that the listed actions have a medium-high impact on ABR (median effectiveness of all actions combined: 5 out of 7 (IQR: 4–6), Figure 6).

**Figure 7.**
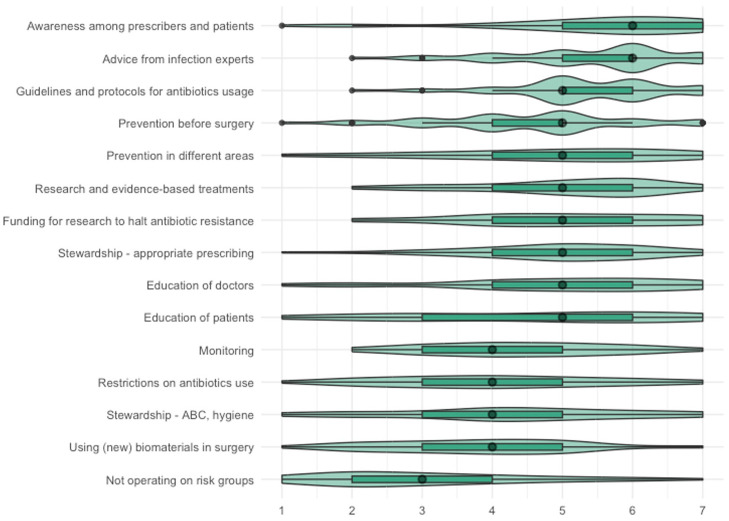
Results of step 3: perceived effectiveness of actions against ABR, actions specified. Very ineffective [1]—very effective [7].

The agreement among participants was high for 14 out of 15 consequences (IQR of size 1–2) and medium for the education of patients (IQR of size 3; Figure 7). Only awareness among prescribers and patients (median: 6; IQR 5–7) and advice from infection experts (median: 6; IQR 5–6) was perceived to be highly effective. Most actions were perceived to have medium-high effectiveness: guidelines and protocols, research (funding), stewardship focused on prescribing, education of doctors and patients, and prevention in different areas (median: 5; IQR between 4 and 6 for 7 items and 3–6 for 1 item). Restrictions on antibiotic use, monitoring, stewardship focusing on hygiene, and the use of biomaterials (median: 4; IQR 3–5) were perceived to have a medium effect. Not operating on risk groups was only perceived to have a medium-low effect (median: 3; IQR 2–4).

For actions that were rated as >4 effective, participants were either asked how confident they were that they could perform that action, or how confident they were that their organization could perform that action. In both conditions, having confidence in an action being performed was more often indicated than not having confidence in that action being performed (60.6% [*n* = 235] and 39.4% [*n* = 153] respectively; Appendix D on OSF: https://osf.io/dkty3/).

## Discussion

This study investigated Dutch orthopedic healthcare professionals’ mental models of ABR to elucidate perceptions that can ultimately inform behavior change strategies. By showing that participants perceived the causes of ABR to be mostly external, the consequences of ABR to be abstract and the most effective actions against ABR to be performed by others, this study forms the basis for understanding the mental model of healthcare professionals regarding ABR. In the first step, key experts were consulted to create the expert model. This model introduced the structure of causes, consequences, and actions related to ABR. In the second step, an open-ended questionnaire was applied. Based on this step, the model was supplemented with specific causes, consequences, and actions, most of which related to healthcare professionals and the hospital. In the third step structured rating questionnaire, the relevance of these causes, consequences, and actions were rated. We conclude that the lists of causes, consequences, and actions used in the third step are complete because, in this step, participants added very few causes, consequences, or actions they felt were missing. Moreover, aggregated ratings from this step indicate that these overall lists of causes, consequences, and actions were perceived as relevant.

Within the categories, the perceived impact of causes and actions and the perceived likelihood of consequences differ. Results on the consequences of ABR show that abstract consequences (e.g. higher costs and increased infections) were perceived as more likely than concrete consequences (e.g. closing wards). The focus on the importance of abstract consequences can be explained by the Construal Level Theory (Trope and Liberman, 2010). This theory posits that abstract thinking (i.e. high construal) can be caused by perceiving a larger psychological distance from a problem (Trope and Liberman, 2010). Previous research indicates that healthcare professionals indeed perceive a large psychological distance from ABR (Harring and Krockow, 2021). ABR is described as a problem that might only occur in a faraway future and faraway countries. This, in turn, relates to the finding of previous studies that healthcare professionals doubt susceptibility to ABR (Krockow et al., 2019; Rose et al., 2021).

Notably, overall, participants for whom the items were framed as consequences for healthcare professionals perceived the consequences of ABR as more likely (5 out 7) than participants for whom the items were framed as consequences for patients (4 out 7). This might point to healthcare professionals being able to imagine consequences for themselves as professionals with more ease than for their patients. Though it might generally be easier to imagine consequences for oneself, it has also been suggested that the consequences of ABR for patients feel more psychologically distant because even patients infected with resistant bacteria can often still be treated with newer antibiotics or combination therapy, limiting the perceived consequences of ABR (Harring and Krockow, 2021).

The actions participants perceived as most effective are often abstract and performed by others. Awareness among prescribers and advice from infection experts were scored as most effective against ABR, and not operating on risk groups was scored as least effective. The preference for more abstract actions might again be a result of the large perceived psychological distance from the problem and the abstract thinking related to it. Indeed, abstract thinking (i.e. high-construal) has been associated with more risky choices (Trope and Liberman, 2010), such as irrational prescribing (Acampora et al., 2023). Moreover, low-construal actions often trigger considering feasibility before deciding to act, whereas, in high-construal actions, desirability influences the decision more (Trope and Liberman, 2010). This may result in orthopedic healthcare professionals focusing less on feasibility and more on desirability when rating the effectiveness of abstract actions, thus rating these actions more positively.

Results on causes of ABR show that external factors such as antibiotic use in other sectors or countries were perceived to contribute to ABR more than internal factors such as local use of antibiotics. Previous qualitative research also indicates that healthcare professionals perceive ABR’s causes as external: overseas, in agriculture, or in the community (Broom et al., 2017). That healthcare professionals perceive to only play a minor role in ABR’s causes does not necessarily mean that they also do not perceive to be able to play a part in a solution. However, in line with abstract consequences being perceived as more likely and with the causes for ABR being perceived as primarily external, the actions participants perceive as most effective are often performed by others. This could suggest that participants perceive to have a limited influence on curbing ABR. That healthcare professionals perceive a limited influence on curbing ABR has also been found in previous studies (Keizer et al., 2019; Krockow et al., 2019).

Notably, risk communication models pose that even when healthcare professionals perceive a high threat of ABR they would need to feel influence on solving this problem before acting. The perceived influence on solving a problem is high in the case of high response efficacy and high self-efficacy (Rogers, 1975). The relatively high perceived effectiveness of actions performed by others could indicate they perceive the actions they perform themselves to have a low response efficacy. Still, participants’ self-reported self-efficacy was relatively high as most participants indicated having confidence in performing most of the listed actions. A possible explanation is that healthcare professionals acknowledge the effect (high response efficacy) of actions performed collectively, but do not recognize the effect (low response efficacy) of actions performed only by them (Harring and Krockow, 2021). They could consider their actions “a drop in the ocean.”

When the scores of all items were aggregated per category (i.e. causes, consequences, actions), the ranges of the aggregated scores were small. The overall scores in one category being close to each other align with the reality that ABR is highly complex, with not one cause, consequence, or action related to ABR having much more or less importance than the others (Ventola, 2015; Wall, 2019). Further in line with this, when looking more closely at the specific perceptions within the categories, no specific causes, consequences, or actions were perceived as extremely likely or effective by all participants (i.e. no median scores were <2 or >6). It is unlikely that the absence of extreme scores is a result of important causes, consequences, or actions being missing from the lists since participants were allowed to add causes, consequences, or actions. We conclude that the absence of extreme scores points to participants perceiving ABR as not having one or a few important causes. Seeing ABR as a complex and wicked problem aligns with previous research which describes that a shared mental model of the problem is lacking (Calvo-Villamañán et al., 2023).

Within the categories, the agreement among the participants for specific perceptions varied. Especially participants’ opinions about perceptions regarding patients differed (i.e. patients using antibiotics without prescription and educating patients). How relevant the participants perceived the role of the patients might follow from how prevalent and serious they perceive patients’ antibiotic misuse and how much pressure to prescribe they feel from patients. Healthcare professionals are the gatekeepers to antibiotics and thus most responsible for antibiotic prescriptions. The public even perceives healthcare professionals to hold this responsibility (Worthington et al., 2020). Still, patients also potentially drive ABR with self-medication and inadequate use of antibiotics (Uddin et al., 2021). Likewise, healthcare professionals perceive patient expectations or pressure to prescribe antibiotics as determinants of antibiotic prescribing and thus of ABR (Acampora et al., 2023; Almeida-Costa et al., 2023). How much pressure to prescribe is perceived differs between healthcare professionals (Almeida-Costa et al., 2023). Future research should investigate if the divergent perceptions about the role of the patient in ABR could be a result of differing perceptions of patients’ antibiotic misuse and the amount of pressure to prescribe applied.

This study has several strengths. Firstly, the qualitative approach to questionnaire development ensured authentic bottom-up input from the target group. Secondly, the expert model and the consecutive rounds confirmed no relevant perceptions related to ABR were missed. Thirdly, the quantitative questionnaire allowed for validation of the previous steps among a larger and more diverse sample of participants. Taken together, this enabled the construction of an accurate, exhaustive, and valid mental model of the perceptions of Dutch orthopedic healthcare professionals. This is essential for understanding their actions against ABR and for developing effective behavior change strategies. Previous studies on the perceptions of healthcare professionals of causes, consequences, and actions related to ABR focused on one aspect of ABR, for example, prescribing (Almeida-Costa et al., 2023; Peeters et al., 2022). These perceptions are too diverse and have been identified in too diverse settings to lead to a coherent overview or mental model (Vonken et al., 2024). Another strength of this study is thus that a specific target group was chosen.

A limitation of this study is that the representativeness of our target group cannot be guaranteed. The field of orthopedics is diverse and despite our efforts only a small target group was recruited. This limits generalizability to other specialties. Further, self-selection of participants interested in ABR might have resulted in selection bias, thereby resulting in overly optimistic results. A further limitation of this study is that changes to the mental model approach were made that have not yet been validated. The method used places more responsibility on the participants and does not allow follow-up questions. The interviews applied in the original mental model approach might have yielded more in-depth information and might have given more insight into individual differences and the relationship between identified perceptions. We do believe that the advantages of the changes made to the mental model methodology outweigh the disadvantages. The questionnaire was answered without interference from a researcher, thereby yielding answers less biased by, for example, social desirability.

This study forms the basis for understanding Dutch orthopedic healthcare professionals’ mental model of ABR. The mental model methodology yielded further insight into perceptions. Still, future research should confirm the constructed model. Firstly, qualitative studies should confirm and further specify the identified perceptions and the relationship between those perceptions. Secondly, qualitative research and experimental studies are suggested to study the link between perceptions, behavioral determinants, and behavior. Thirdly, quantitative studies should study whether the findings can be transferred to other specialties, sectors, and countries. The participants in this study were all Dutch healthcare professionals. The Netherlands is one of the frontrunners in restrained antibiotic prescribing (European Centre for Disease Prevention and Control, 2023). Correspondingly, Dutch healthcare professionals perceive ABR as a problem more relevant in other countries and sectors (Vonken et al., 2024). Fourthly, quantitative studies, applying for example cluster analysis, should also determine whether clusters of participants with similar perceptions exist. For example, current results suggest some healthcare professionals recognize the strong role of patients in the development of ABR and some do not. Clusters with different perceptions should be targeted differently in interventions.

A detailed and established mental model allows for developing and tailoring interventions to change perceptions and behaviors, making it more likely that these interventions will be effective (Noar et al., 2007; Sleboda and Lagerkvist, 2022). Actionable findings from this study are that ABR is perceived as multifactorial and healthcare professionals disagree on the role of the patient in ABR. Unifying communication about ABR and consistently focusing on selected main messages can help form a shared mental model with special attention to the most important causes, consequences, and actions. For example, communication should focus on unifying the differing opinions about the role of the patient. Behavior change initiatives are recommended to aim to decrease the psychological distance of ABR. We know that when someone perceives the risks of an action as more proximal and concrete, one is more likely to act responsibly (Maiella et al., 2020). In addition, behavior change initiatives should aim to increase perceived susceptibility to ABR and response efficacy for curbing ABR.

### Conclusions

Dutch orthopedic healthcare professionals perceive the causes of ABR to be mostly external, the consequences of ABR to be abstract and the most effective actions against ABR to be performed by others. No specific causes, consequences, or actions are perceived to strongly outweigh the others. Perceptions differ about the role of the patient in the progression of ABR, for example, about what the impact of patients using antibiotics without a prescription is. Unified communication about ABR is needed to highlight causes, consequences, and actions most relevant to healthcare professionals. To encourage action against ABR among healthcare professionals, behavior change initiatives should be applied to decrease the perceived distance from ABR, increase perceived susceptibility to ABR, and increase response efficacy in curbing ABR. Ultimately, this can help tackle the impact of infections and ABR in orthopedics.

## Supplemental Material

sj-docx-1-hpq-10.1177_13591053251332101 – Supplemental material for How Dutch orthopedic healthcare professionals perceive antibiotic resistance: A mixed-methods application of the mental model approach Supplemental material, sj-docx-1-hpq-10.1177_13591053251332101 for How Dutch orthopedic healthcare professionals perceive antibiotic resistance: A mixed-methods application of the mental model approach by Lieve Vonken, Gert-Jan de Bruijn, Stef Kremers and Francine Schneider in Journal of Health Psychology

sj-pdf-2-hpq-10.1177_13591053251332101 – Supplemental material for How Dutch orthopedic healthcare professionals perceive antibiotic resistance: A mixed-methods application of the mental model approach Supplemental material, sj-pdf-2-hpq-10.1177_13591053251332101 for How Dutch orthopedic healthcare professionals perceive antibiotic resistance: A mixed-methods application of the mental model approach by Lieve Vonken, Gert-Jan de Bruijn, Stef Kremers and Francine Schneider in Journal of Health Psychology

sj-pdf-3-hpq-10.1177_13591053251332101 – Supplemental material for How Dutch orthopedic healthcare professionals perceive antibiotic resistance: A mixed-methods application of the mental model approach Supplemental material, sj-pdf-3-hpq-10.1177_13591053251332101 for How Dutch orthopedic healthcare professionals perceive antibiotic resistance: A mixed-methods application of the mental model approach by Lieve Vonken, Gert-Jan de Bruijn, Stef Kremers and Francine Schneider in Journal of Health Psychology
