# Supplementary material for: How Dutch orthopedic healthcare professionals perceive antibiotic resistance: A mixed-methods application of the mental model approach
Source: J Health Psychol. 2025 Apr 28;30(14):4494–512. doi: 10.1177/13591053251332101 (PMC12678642; doi:10.1177/13591053251332101)
Supplement: sj-docx-1-hpq-10.1177_13591053251332101 – Supplemental material for How Dutch orthopedic healthcare professionals perceive antibiotic resistance: A mixed-methods application of the mental model approach [file sj-docx-1-hpq-10.1177_13591053251332101.docx]

**Appendix A**


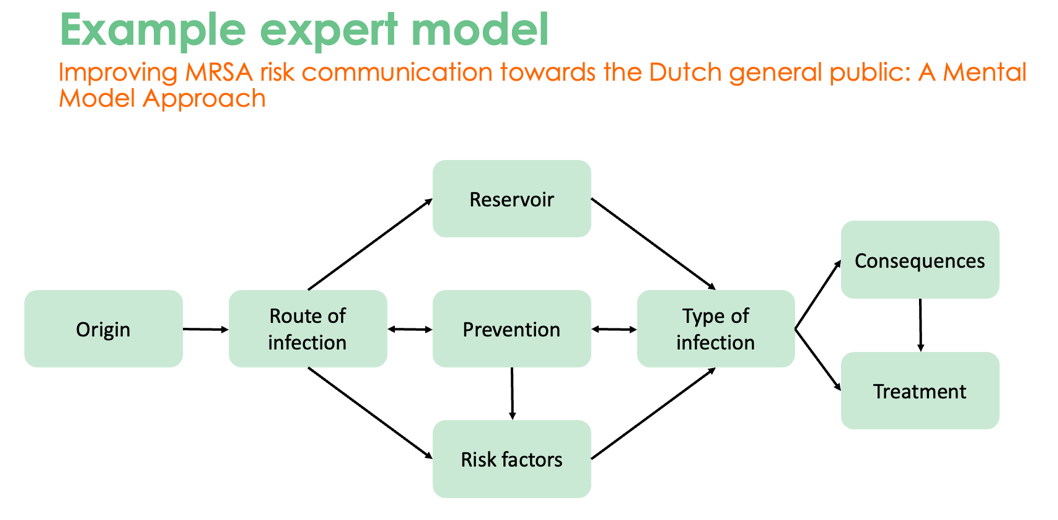

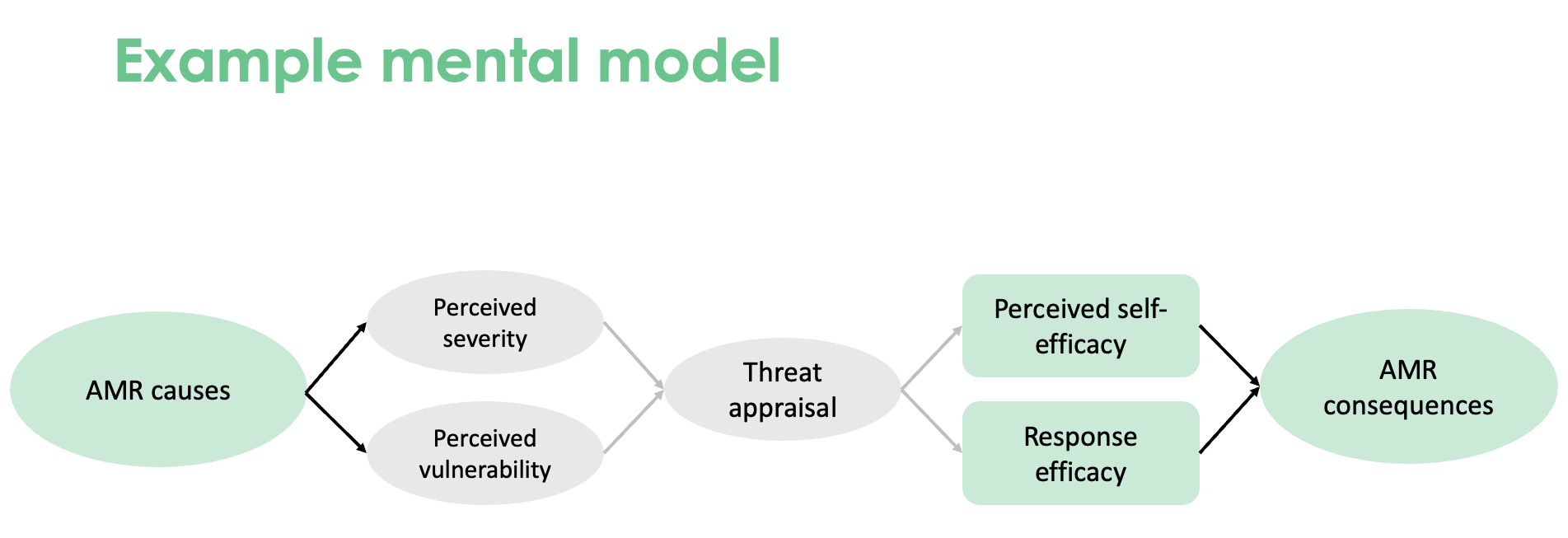

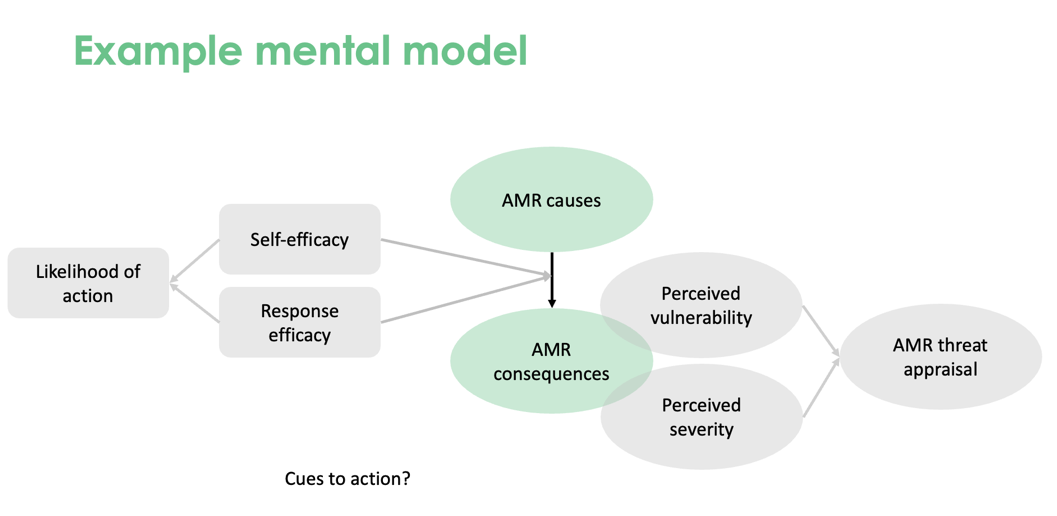


Adapted from: Reijerink, J. (2017). *Improving MRSA risk communication towards the Dutch general public: A Mental Model Approach*(Master's thesis, University of Twente).
