# Supplementary material for: How Dutch orthopedic healthcare professionals perceive antibiotic resistance: A mixed-methods application of the mental model approach
Source: J Health Psychol. 2025 Apr 28;30(14):4494–512. doi: 10.1177/13591053251332101 (PMC12678642; doi:10.1177/13591053251332101)
Supplement: sj-pdf-2-hpq-10.1177_13591053251332101 – Supplemental material for How Dutch orthopedic healthcare professionals perceive antibiotic resistance: A mixed-methods application of the mental model approach [file sj-pdf-2-hpq-10.1177_13591053251332101.pdf]

Table 1. Characteristics of participants excluded in step 3: job context, ABR experience and felt urgency of ABR.

**Hospital type**			**Job description/main purpose**			**How often they work on ABR**		
	**N**	**%**		**N**	**%**		**N**	**%**
Community hospital	10	76.4	Medical specialists	6	54.6	Daily	3	27.3
			Nurses	5	45.5	Weekly	3	27.3
Academic hospital	1	21.8	Residents	0	0	Monthly	3	27.3
			Doctors not in residency	0	0	Half-yearly	1	9.1
			Researchers	0	0	Less than half-yearly	1	9.1

**ABR expert**			**ABR urgency**		
	**N**	**%**		**N**	**%**
Strongly disagree	0	0	Not at all urgent	0	0
Disagree	2	18.2	Not urgent	0	0
Somewhat disagree	3	27.3	Somewhat not urgent	0	0
Neither agree nor disagree	1	9.1	Neutral	1	9.1
Somewhat agree	4	36.4	Somewhat urgent	3	27.3
Agree	1	9.1	Urgent	7	63.6
Strongly disagree	0	0	Very urgent	0	0

Years of experience:
Mean: 12 years
Range: 1-38 years
Standard deviation: 12.2 years

Table 2. Output of Fischer's Exact tests comparing included and excluded participants of step 3.

**Variable**	**Test statistic**	**Significance**
Hospital type	1.252	.532
Job description/main purpose	5.867	.291
How often they work on ABR	3.511	.466
ABR expert	7.964	.184
ABR urgency	4.086	.258

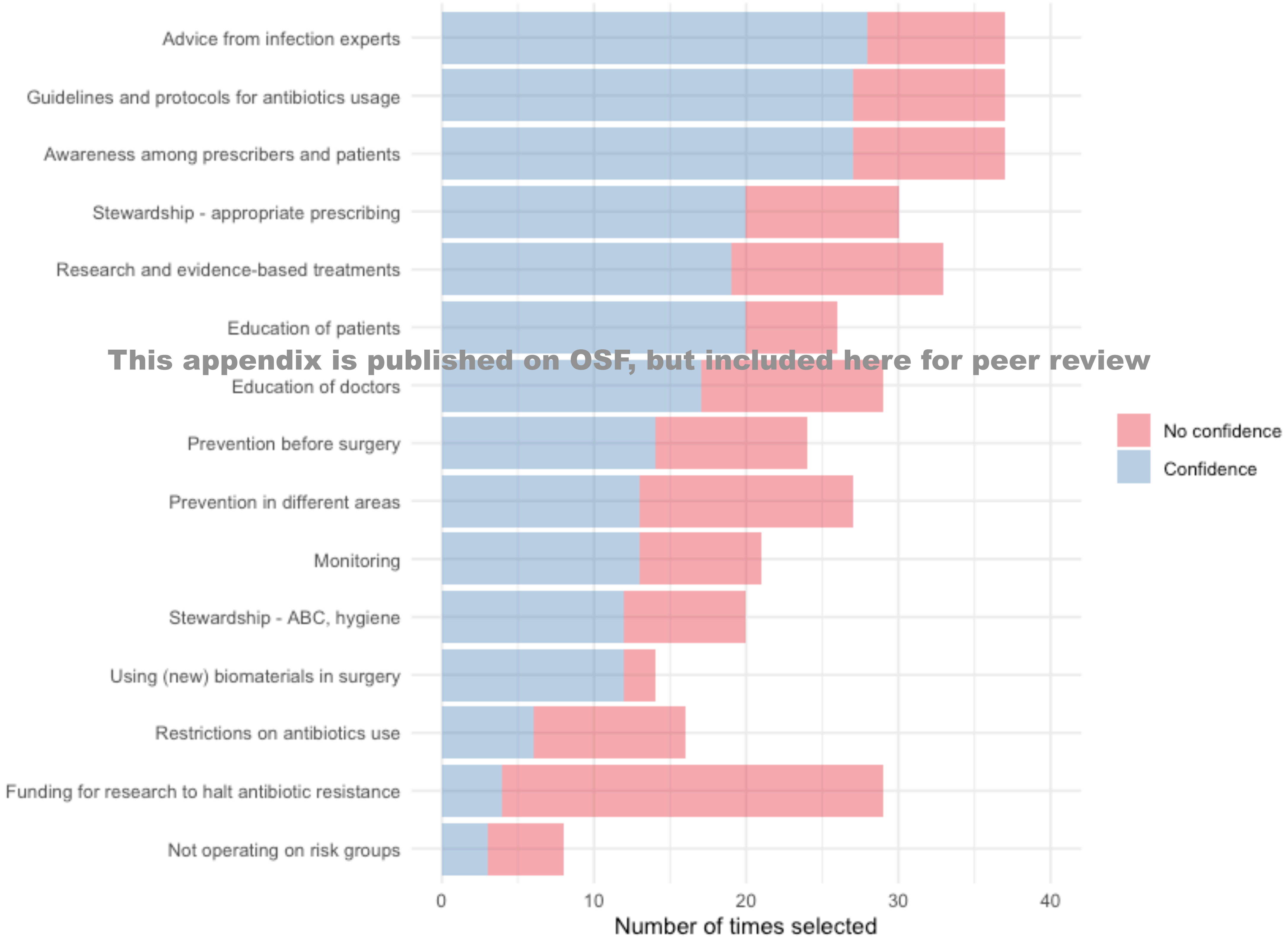

This appendix is published on OSF, but included here for peer review

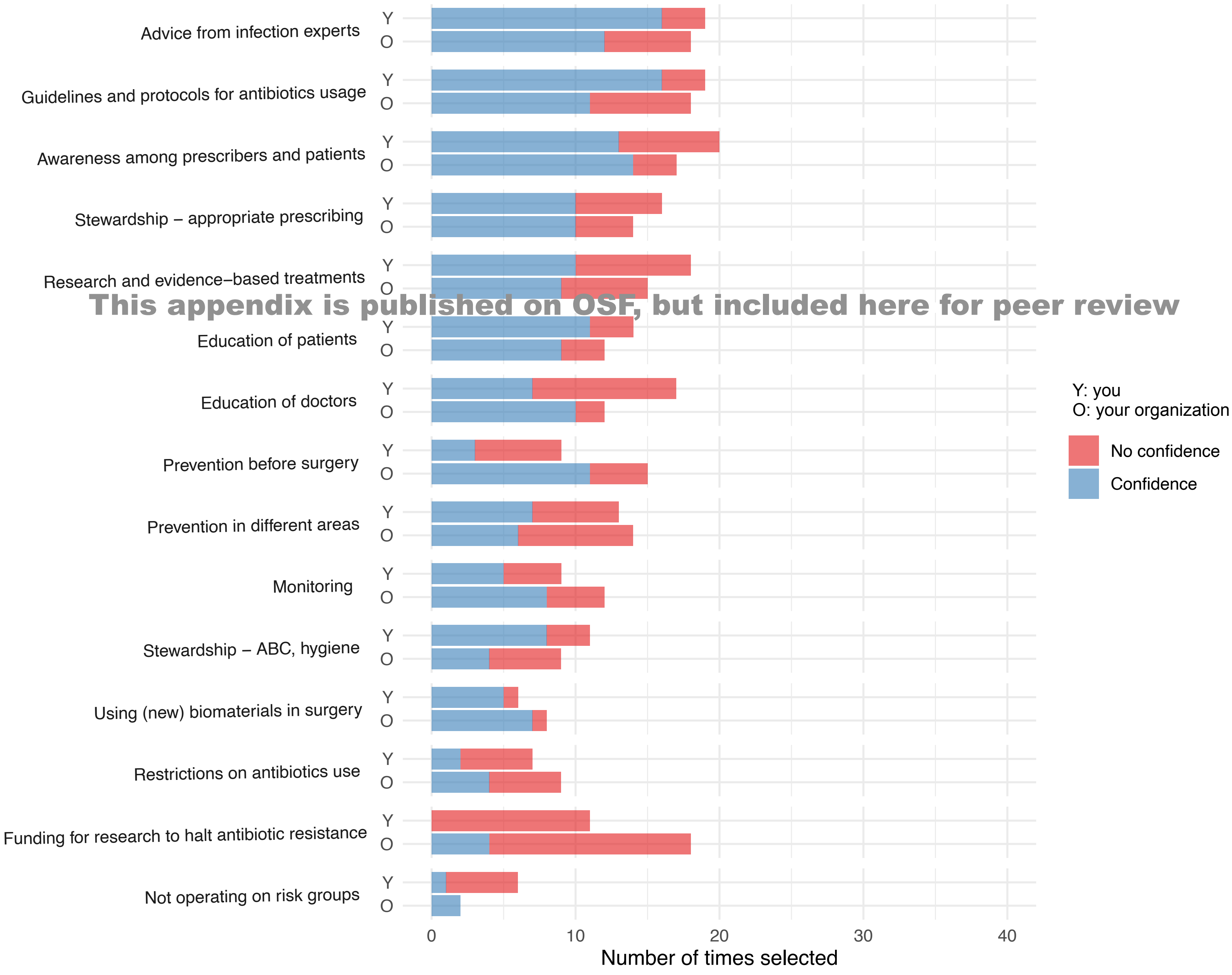

This appendix is published on OSF, but included here for peer review

This appendix is published on OSF, but included here for peer review

Perceived confidence in actions against ABR.

	Confidence	No confidence	Total
You	114 (58.5%)	81 (41.5%)	195 (100%)
Organization	121 (62.7%)	72 (37.3%)	193 (100%)
Total	235 (60.6%)	153 (39.4%)	388 (100%)
