# Supplementary figures and images for: How Dutch orthopedic healthcare professionals perceive antibiotic resistance: A mixed-methods application of the mental model approach
Source: J Health Psychol. 2025 Apr 28;30(14):4494–512. doi: 10.1177/13591053251332101 (PMC12678642; doi:10.1177/13591053251332101)

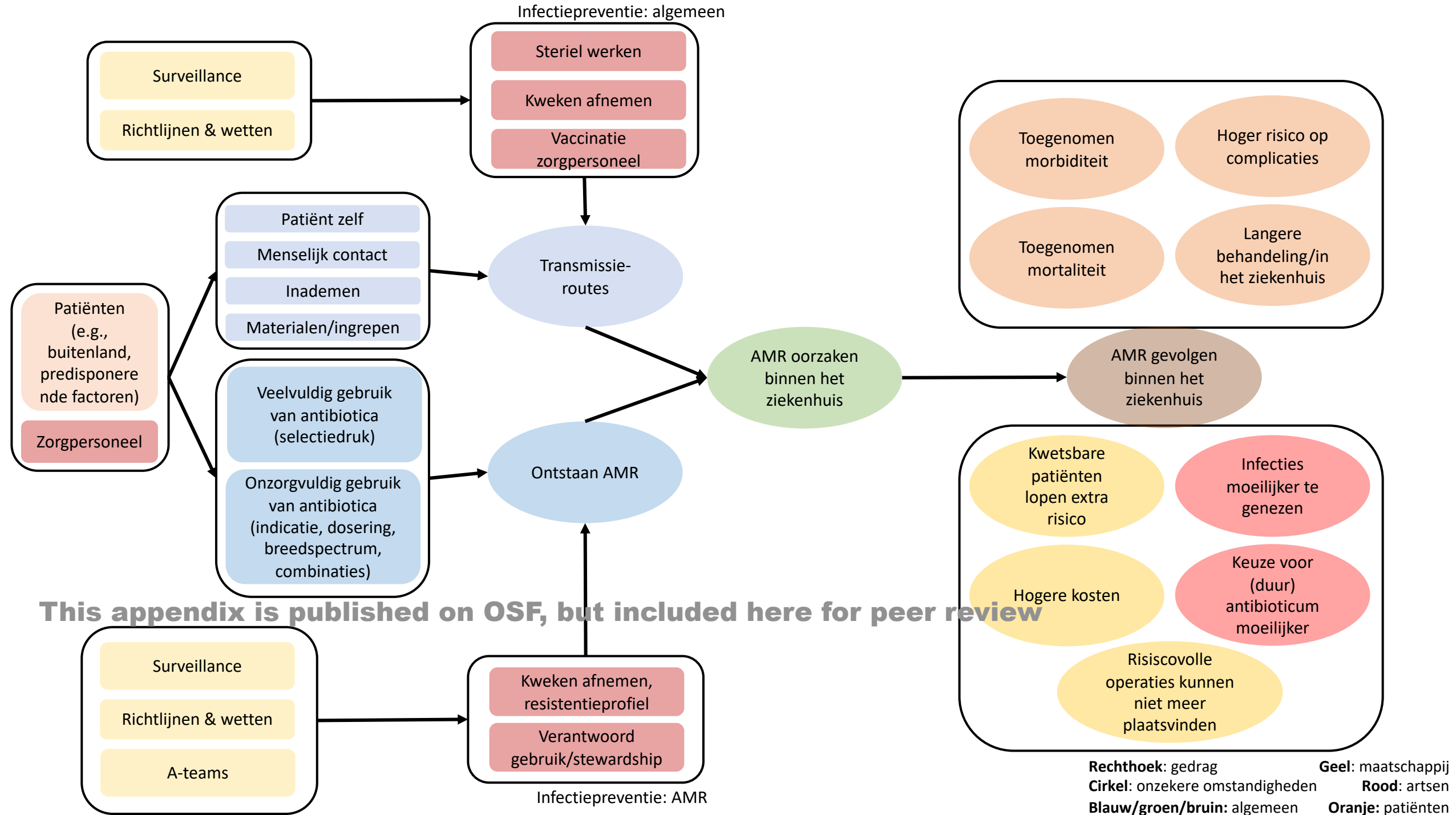

Supplement: sj-pdf-3-hpq-10.1177_13591053251332101 – Supplemental material for How Dutch orthopedic healthcare professionals perceive antibiotic resistance: A mixed-methods application of the mental model approach [file sj-pdf-3-hpq-10.1177_13591053251332101.pdf]
